# Intestinal Microbiota Confer Protection by Priming the Immune System of Red Palm Weevil *Rhynchophorus ferrugineus* Olivier (Coleoptera: Dryophthoridae)

**DOI:** 10.3389/fphys.2019.01303

**Published:** 2019-10-16

**Authors:** Abrar Muhammad, Prosper Habineza, Tianliang Ji, Youming Hou, Zhanghong Shi

**Affiliations:** ^1^State Key Laboratory of Ecological Pest Control for Fujian and Taiwan Crops, Fujian Agriculture and Forestry University, Fuzhou, China; ^2^Fujian Provincial Key Laboratory of Insect Ecology, College of Plant Protection, Fujian Agriculture and Forestry University, Fuzhou, China

**Keywords:** gut microbiota, *Rhynchophorus ferrugineus*, insect immunity, invasive biology, symbiosis

## Abstract

The immune system of animals, including insects, is the vital factor to maintain the symbiotic interactions between animals and their associated microbes. However, the effects of gut microbiota on insect immunity remain mostly elusive. Red palm weevil (RPW), *Rhynchophorus ferrugineus* Olivier, is a destructive pest of palm trees worldwide, which has forged alliances with its gut microbiota. Here, we found that the aposymbiotic insects succumbed at a significantly faster rate than conventionally reared (CR) ones upon bacterial infection. Physiological assays confirmed that CR insects had stronger antimicrobial activity and higher phenoloxidase activity in contrast to germfree (GF) ones, indicating that the systemic immune responses of GF individuals were compromised markedly. Interestingly, under the bacterial challenge conditions, the reassociation of gut microbiota with GF insects could enhance their survival rate by rescuing their immunocompetence. Furthermore, comparative transcriptome analysis uncovered that 35 immune-related genes, including pathogen recognition receptors, effectors and immune signaling pathway, were significantly downregulated in GF insects as compared to CR ones. Collectively, our findings corrobate that intestinal commensal bacteria have profound immunostimulatory effects on RPW larvae. Therefore, knowledge on the effects of gut microbiota on RPW immune defenses may contribute to of set up efficient control strategies of this pest.

## Introduction

As in vertebrates, diverse groups of microbes, being generally known as gut microbiota, also colonize insect guts. Gut microbiota has been attested to profoundly affect host physiological traits including nutrition provision (Wong et al., 2014; Janssen and Kersten, 2015), growth and development (Blatch et al., 2010; Wong et al., 2014), mating and foraging behavior (Ami et al., 2010), and gut homeostasis (Engel and Moran, 2013; Douglas, 2015). Considering both pathogenic and beneficial bacterial species possess the conserved immune elicitors, it is the dilemma that insect hosts should preserve the beneficial microbes while ward off pathogenic intruders by mounting effective, yet not damaging defense responses. Accumulating evidence has indicated that some physical barriers, such as peritrophic matrix and mucus layer (Martens et al., 2018), and gut immunity play crucial roles in maintaining the homeostasis of gut microbiota (Lee et al., 2017; Dawadi et al., 2018). For example, gut epithelial cells can secrete reactive oxygen species (ROS) and antimicrobial peptides to regulate the intestinal microbiota in *Drosophila melanogaster* (Ha et al., 2005; Ryu et al., 2008; Lee et al., 2017). To our knowledge, an increasing number of observations have shown that endosymbiotic bacteria, such as *Wolbachia* and *Wigglesworthia* in tsetse flies and *Buchnera* in aphids, can confer resistance to host against the natural enemies (Oliver et al., 2007; Weiss et al., 2012; Johnson, 2015). Unfortunately, the effects of gut microbiota on the development and proper presence of insect immunity and the mechanisms underlying gut microbiota-induced impacts on host immunity are far from being well understood, especially for the invasive insect pests.

Red palm weevil (RPW), *Rhynchophorus ferrugineus* Olivier, is a destructive pest for palm trees worldwide (Ju et al., 2011; Wang et al., 2015). In China, it has killed almost 20,000 coconut palms in the area of over 10,000 km^2^ as an invasive pest since 1997 (Li et al., 2009; Ju et al., 2011; Ge et al., 2015). RPW complete its development from the egg to newborn adult in the trunk of palm trees and the weevil larva is the major infestation stage which feed on tender tissues from the apical growing point of palm trunk (Butera et al., 2012; Shi et al., 2014). Because RPW always live inside palm trunks except for copulation and oviposition, it is usually difficult to find RPW adults and larvae, let alone trying to kill them with chemical pesticides. Consequently, it is urgent to develop integrated pest management tactics based on pheromone traps and biological agents. However, current evidences have indicated that the control efficiency of biological agents is always limited. It is widely acknowledged that insects solely rely on a suite of innate immune responses, comprising cellular and humoral defense mechanisms, to fight pathogenic microbes (Shi et al., 2014). Presently, RPW has been found to live in the symbiotic associations with gut microbiota which is involved in its nutrition metabolism (Muhammad et al., 2017; Prosper et al., 2019). To our knowledge, it is still unknown about if and how commensal microbiota is involved in the proper immune function of insects outside dipteran insects. Because insects often combat pathogens by triggering their innate immune responses, investigations on the intimate relationships between RPW immunity and its gut microbiota are very promising via providing new insights to improve and even drive the development of novel control tactics.

In this study, the influence of commensal microbiota on RPW immunity was determined by comparing the immunocompetence and survival ability of GF and CR RPW larvae upon pathogenic bacterial challenge. Furthermore, transplanting gut microbiota to GF larvae by introducing gut homogenate from CR ones on diet was executed to evaluate whether the compromised immunity phenotypes in GF larvae could be restored. Finally, comparative transcriptome analysis on the immunity of RPW CR and GF larvae was applied to uncover the possible molecular mechanisms behind intestinal bacteria-induced maturation of host immunity. The data we presented here reinforce the understanding of how commensal microbiota affect RPW immunity and set the groundwork for further investigation on the interplay of RPW gut symbionts with the host immune system.

## Materials and Methods

### Insect Rearing and Generation of Germfree Individuals

Red palm weevil insects were maintained in our laboratory at 27 ± 1°C and 75 ± 5% relative humidity. Larvae were reared in a photoperiod of 24 h dark on artificial diet (Pu et al., 2017), while adults were maintained in climatic chambers as described previously (Muhammad et al., 2017). Treatment cohorts of RPW in this study were designated in four groups: (I) Conventionally reared (CR) larvae contained their inherent gut microbiota; (II) Germfree (GF) ones, being completely devoid of gut microbiota, were generated with the following steps: RPW eggs were washed in 10% sodium hypochlorite solution (NaClO) for 3–5 min, rinsed in 75% ethanol two times to remove the bacteria on the egg surface, and then rinsed with sterilized distilled water two times to exclude the potential effect of sodium hypochlorite and ethanol on the following experiments. The neonatal larvae were fed on the sterile diet (date palm tissue 8.0 g, sucrose 8.0 g, agar 6.0 g, casein 8.0 g, corn flour 10.0 g, yeast extract 12.0 g, avicel 5.0 g, ascorbic acid as vitamin C 1.0 g, potassium sorbate 0.4 g, sodium *p*-hydroxybenzoate 0.2 g, cholestrol 0.3 g, choline chloride 0.25 g, inositol 0.02 g, and 220 ml distilled water) with antibiotic cocktail (Kanamycin, Tetracycline, Gentamycin, and Erythromycin, the final concentration of 600 mg/L) in the laminar hood (Heal Force safe-1200LC, China). (III) GF + BasSup larvae were established by feeding the gut homogenate from CR larvae to GF individuals (Supplementary Figure S1), and (IV) CR Dech larvae, being from dechorionated eggs and reared on sterile food without antibiotics, contained impaired (≈80% less) gut microbiota (Supplementary Figure S1). GF larvae were always maintained on the food added with antibiotics, but CR Dech. and CR feed on the diet without antibiotics. The 3rd instar GF + BacSup. larvae were provided with food without antibiotics until they were used in the experiments. The procedures on how to generate and maintain these GF and GF + BasSup larvae have been described in detail by Prosper et al. (2019).

### Bacteria Preparation for Infection Assays

*Serratia marcescens* strain RPWL1 (Accession No. MF185369) was previously isolated and identified from RPW gut (Pu and Hou, 2016; Muhammad et al., 2017) and eGFP-tagged *Escherichia coli* was prepared in LB (Luria-Bertani) medium at least 2 days prior to infection according to the steps of Dawadi et al. (2018). The OD_600_ (optical density) value of *S. marcescens* and *E. coli* solution was 1.89 and 1.00, respectively. All assays were repeated at least three times.

### Determination of Phenoloxidase (PO) Activity

To measure the PO response of symbiotic and aposymbiotic individuals and the effect of bacterial infection on the PO activity, the symbiotic (harboring gut microbiota, i.e., CR and GF BacSup.) and aposymbiotic (GF) fourth instar larvae were challenged with *S. marcescens*. Systemic infection was established by injecting 3 μl (OD_600_ = 1.89) *S. marcescens* solution directly into the hemocoel using a 10 μl glass syringe (Hamilton). Parallel controls were established by injecting the same volume of sterile PBS. Before being injected, larvae from different cohorts were chilled on ice for 5–7 min to be immobilized and then their body surface were sterilized in 75% ethanol and washed with sterile water. Six hours post infection, the hemolymph was collected from the larvae as described previously (Muhammad et al., 2017). Hemolymph samples were centrifuged for 5 min at 8000 *g* (4°C) and then the supernatant was collected into a new tube. The PO activity was assayed using SpectraMax (Molecular Devices, United States) spectrometer according to the methods described by Meng et al. (2016).

### The Assays on the Ability of Bacteria Clearance in Hemolymph

To investigate the antimicrobial activity of symbiotic and aposymbiotic RPW individuals, the 4th instar larvae were challenged with 10 μl eGFP-tagged *E. coli* solution as described above. The same volume of sterile PBS was injected into the same instar larvae as control. Bacterial load in hemolymph was assayed at 6 hpi (hour post-infection) and the hemolymph samples were collected with the above procedures. 100 μl hemolymph samples were spread on the LB agar plates with ampicillin (100 μg/ml) which were incubated for 18 h at 37°C. The CFUs were counted by visualizing the plates under Leica MZFII Stereo Fluorescence Microscope (Leica Microsystems, Germany). Three larvae were used in this assay from each cohort and each treatment was replicated for four times.

### Septic Infection and Survival Assay

To determine whether gut microbiota benefit host survival, the symbiotic and aposymbiotic fourth instar larvae were challenged by *S*. *marcescens* as described in PO activity assays. The injected larvae were maintained in a separate growth chamber and the number of live or dead larvae was counted every 12 h to monitor insect’s survival. This experiment was repeated three times and 10 larvae were treated in each group of every replicate.

### Transcriptome Analysis on Gut Immunity of CR and GF Larvae

To detect the microbiota-induced changes in gut immunity, comparative RNA-Seq analysis on the guts of the fourth instar CR and GF RPW larvae was applied. Whole guts were dissected in sterile conditions for RNA extraction as described previously (Muhammad et al., 2017). Separate libraries, being constructed from total RNA samples of CR and GF groups, were sequenced on Illumina HiSeq^TM^ 4000 system (Guangzhou Gene Denovo Biotechnology Co., Ltd., China). *de novo* assembly of transcriptome was performed with Trinity software package. For functional annotation, the unigenes were searched against four databases (Nr, Swissport, KEGG, and COG/KOG) using BLASTx program^1^ with an *E*-value of 10^–5^ cut off threshold. Gene Ontology (GO) analysis was completed with Blast2GO software according to the GO association with Nr annotation results of unigenes. Differential expression analysis between the gut transcriptome of CR and GF insects was executed using a rigorous algorithm. Based on the IDs of previously identified immune genes, putative immunity-related genes from RPW gut transcriptome were searched manually against the immune genes of insects such as *Tribolium castaneum* (Zou et al., 2007), *Locusta migratoria manilensis* (Zhang et al., 2015), and *Ostrinia furnacalis* (Liu et al., 2014). The accession number of our sequence data is SAMN11959955-11959960.

### The Impact of Gut Bacteria on the Transcript Abundance of Immunity-Related Genes Validated by RT-qPCR

To validate the intestinal-induced expression level of some important immune genes, 15 genes from four functional categories, i.e., pathogen recognition receptors, signal modulation, signal transduction and immune effectors, were selected for transcript quantification. Among these genes, PGRPs (*PGRP-LB, PGRP-LC, PGRP-LE*, and *PGRP-S1*), *Relish, Defensin, Coleoptericin, Attacin*, and *Cecropin* have been cloned and verified by us. while *Beta-glucosidase, C-type lectin (CTL), EF-hand domain containing protein, Serine protease like protein, C-type lysozyme*, and *Cathepsin* were determined according to the previously published data (Hussain et al., 2016). Three individual guts were pooled as a replicate and TRIzol Reagent (Invitrogen, United States) were used to extract total RNA from these gut samples following the manufacturer’s protocol. Each treatment was independently repeated with at least three times. RNA integrity, quality, and concentrations were assessed by Nanodrop (Thermo scientific 2000) and electrophoresis on 1% agarose gel. Total RNA samples were then stored at −80°C until further use. TransScript^®^ All-in-One First Strand cDNA Synthesis Kit (Cat# AT341) was used for synthesizing cDNA. cDNA was serially diluted (5, 25,125, 625, and 3125 fold) to generate standard curves for the target genes to determine primers efficiency and optimal template concentration. RT-qPCR analysis was conducted to determine the fold changes of genes. The qPCR reaction system (20 μl) comprised 10 μl FastStart Universal SYBR Green Master Mix (ROX, Roche) (Life Technologies, United States), 1 μl cDNA, and 3 μM of each primer. Primers used in this study are listed in Table 1. Reactions were performed in triplicate in 96 well plates on a 7500 Fast Real Time PCR system (Applied Biosystem, Life Technologies) with the following thermal program: denaturation at 95°C for 10 min, 40 cycles of 95°C for 15 s, and amplification at 60°C for 60 s and with a dissociation step.

**TABLE 1:** The specific primers used for RT-qPCR analysis.

**Target gene**	**Forward Primer (5′-3′)**	**Reverse Primer (5′-3′)**
*RfPGRP-LC*	TGCACATCCCACGCTAAATG	ATAGCTCCATCGCCACCTAC
*RfPGRP-LB*	TACGGAATGCCCTGGTGATG	GTGGGATCTGCTACCCAGTG
*RfPGRP-LE*	GTGTCGGATGTGATGGAAATG	GCTGAGGAGGAAGCTTGTTG
*RfPGRP-S1*	TTTCGTCGCTCTGTTCGCTATT	TCGTGGTACTTAGGGCTGGTC
*Rf*β*-glucosidase*	TATGGCATGGGCCTTGACTG	GGTGTTCTCGGTCTCTCTGG
*RfC-type lectin*	TGGTACTCCACGCCATCAAC	ATCAGCTACCCACTTTCCGC
*RfRelish*	GAAGTATGGGAAGGATGGGGT	GGACTGGTTGTGTAATGTTCGAG
*RfSerine-protease*	TTTGTCTGACCGCACCAAGT	TACCGAGCACCATCCACAAC
*RfEF-hand domain protein*	CCAACTGATGGACCACGACA	CTCGTTGGCGATCTTACCGA
*RfAttacin*	TGGTTCTGGTGCCCAAGTGA	GCCATAACGATTCTTGTTGGAGTA
*RfCecropin*	CAGAAGCTGGTTGGTTGAAGA	GCAACACCGACATAACCCTGA
*RfDefensin*	TTCGCCAAACTTATCCTCGTG	GGGTGCTTCGTTATCAACTTCC
*RfColeopterecin*	TCGTGGTTTCTACCATGTTCACT	TCAGCTAAAACCTGATCTTGGA
*RfC-type lysozyme*	TAGCACACCAGGCAAAGGTT	TTCGTTGATCCCTTGGCAGT
*RfCathepsin*	GCCCCTACTCCTTGAACCAC	CCACCCCAGGAGTTCTTGAC
*Rf*β*-actin*	CCAAGGGAGCCAAGCAATT	CGCTGATGCCCCTATGTATGT

### Expression Analysis of Immunity-Related Genes in Fat Body in Symbiotic and Aposymbiotic Individuals After Bacterial Challenge

To investigate the effects of gut microbiota on the systemic immune response in symbiotic and aposymbiotic individuals, they were challenged with *S. marcescens* as mentioned above. The fold changes of the immunity-related genes, containing *RfPGRP-LB, RfPGRP-LC, RfPGRP-LE*, *RfPGRP-S1, RfRelish, RfDefensin, RfColeoptericin, RfAttacin*, and *RfCecropin*, were determined by RT-qPCR. 6 h after bacterial challenge, the specimens were dissected for collecting fat body that was subsequently processed for RNA extraction. RNA extraction, cDNA synthesis and RT-qPCR analysis assays were performed as described above.

### Statistical Analysis

One-way ANOVA or Independent-Sample *t*-test was used to detect the statistical differences. All statistical analyses were finished using IBM SPSS Statistics (V. 22.0) and the significance was determined at *P* < 0.05. Kaplan Meir log-rank test was performed to determine if significance between survival rate of symbiotic and aposymbiotic individuals after bacterial challenge.

## Results

### The Commensal Microbiota Deprival Compromised the Immunocompetence and Survival Rate of RPW Larvae

**FIGURE 1:**
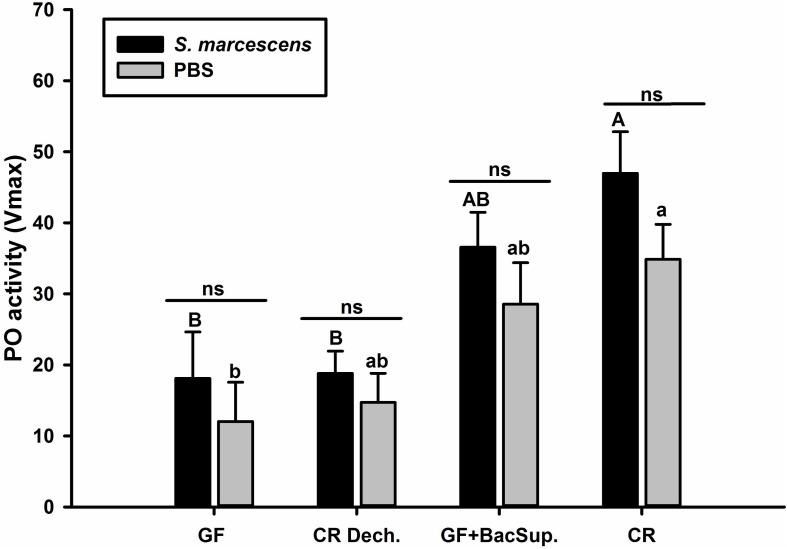
The Phenoloxidase (PO) activity of symbiotic (CR Dech., GF BacSup., and CR) and aposymbiotic (GF) RPW larvae. Uppercase letters indicate the statistical differences of PO activity across different PBS injection groups, whereas the lowercase letters represent the significance of PO activity across different bacterial infection groups using *post hoc* multiple comparisons Tukey’s HSD (Honest Significant Difference, *P* < 0.05). Pairwise comparison for each cohort was performed between infected and control counterparts with independent *t-*test to reveal the effect of bacteria challenge on PO activity. Error bars represent the standard deviation of five independent replicates (ns, *P* > 0.05).

**FIGURE 2:**
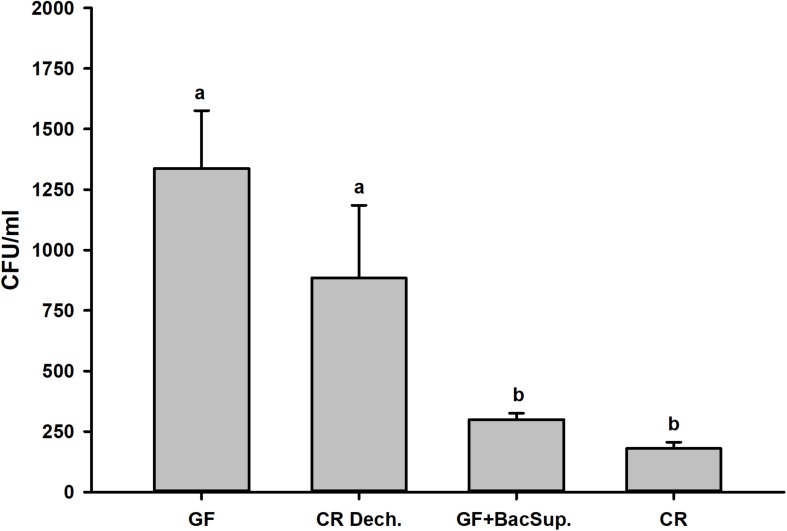
Bacterial clearing ability of the symbiotic (CR Dech., GF BacSup., and CR) and aposymbiotic (GF) RPW larvae.

**FIGURE 3:**
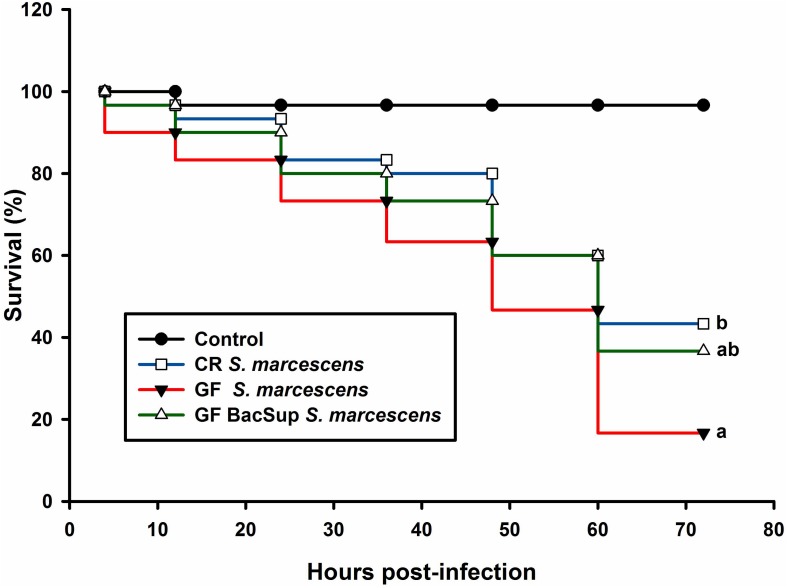
Red palm weevil survival rate of RPW larvae after *Serratia marcescens* infection. The control (black line) insects were injected with sterile PBS. The symbiotic (CR, blue line and GF BacSup., green line) and aposymbiotic (GF, red line) individuals were injected with *Serratia marcescens*. Different alphabetic letters represent the significance (Kaplan Meier Survival analysis, Log rank test *P* < 0.05).

Significant differences were detected in PO activity of RPW larvae from four treated groups without infection (ANOVA: *F*_(__3__,__16__)_ = 4.54, *P* < 0.05) or following the bacterial challenge (ANOVA: *F*_(__3__,__16__)_ = 7.19, *P* = 0.003). As shown in Figure 1, PO activity of CR individuals was markedly higher than that of GF ones. To further uncover the effects of gut microbiota on the antimicrobial activity of this pest, the ability of RPW larvae to clear the injected bacteria from its hemocoel was measured. 1337.45 ± 478.07 CFU of eGFP-tagged *E. coli* were harvested from the hemolymph of GF insects, which was ten folds greater than that of CR ones. However, less CFU of eGFP-tagged *E. coli* were found in the symbiotic insects (CR and GF BacSup) when compared to GF ones, indicating that they had higher pathogen clearance ability than aposymbiotic (GF) or depleted (CR Dech.) symbiotic insects (ANOVA: *F*_(__3__,__12__)_ = 22.10, *P* < 0.001). Interestingly, no statistical significance was detected in the CFU number between CR and GF BacSup groups, suggesting that the reassociation of gut bacteria with GF larvae could rescue their pathogen clearance ability significantly (Figure 2). After being challenged by *S*. *marcescens*, GF larvae died at a significantly higher rate as compared to CR ones (Kaplan Meier Survival analysis, *P* < 0.05, Figure 3). The corrected mortality of GF BacSup was 62.22 ± 3.84% which was lower than that of GF ones (83.33 ± 15.27%). Collectively, these findings support that gut microbiota of RPW can stimulate the immune system against septic pathogen infection. No obvious differences were determined in the PO, antimicrobial activity and survival percentage of CR and GF Bacsup insects, indicating that introducing commensal bacteria into the gut of GF insects could improve their immunocompetence significantly.

### The Induced Higher Expression Level of the Immunity-Related Genes by the Intestinal Bacteria in RPW Larvae

**FIGURE 4:**
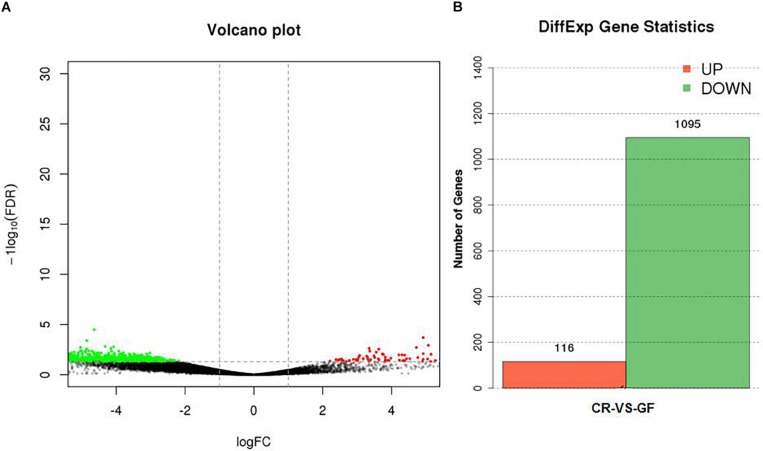
The effect of gut microbiota on the expression level of unigenes in the guts of RPW larvae **(A)** and the number of genes with significant differences in transcript abundance between CR and GF guts **(B)**. Green means downregulation, red means upregulation and black means no significance.

**FIGURE 5:**
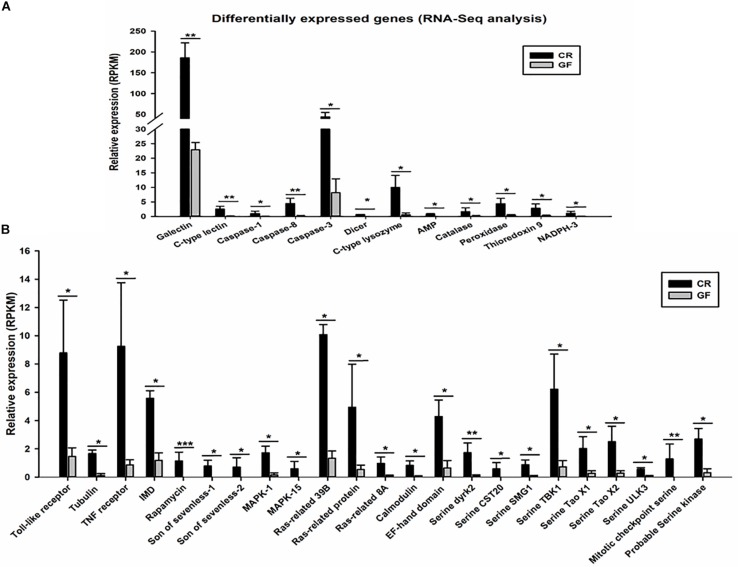
Expression profile of differentially expressed immune related genes (35 genes). RNA-Seq analysis identified immune recognition, immune effectors, and other immune genes **(A)** and immune signaling genes **(B)** significantly downregulated in the guts of GF RPW larvae. The False Discovery Rate (FDR) value was set at *P* < 0.05 and twofold change only for the unigene to be considered as differently expressed genes (DEGs). ^∗^*P* < 0.05, ^∗∗^*P* < 0.01, ^∗∗∗^*P* < 0.001.

**FIGURE 6:**
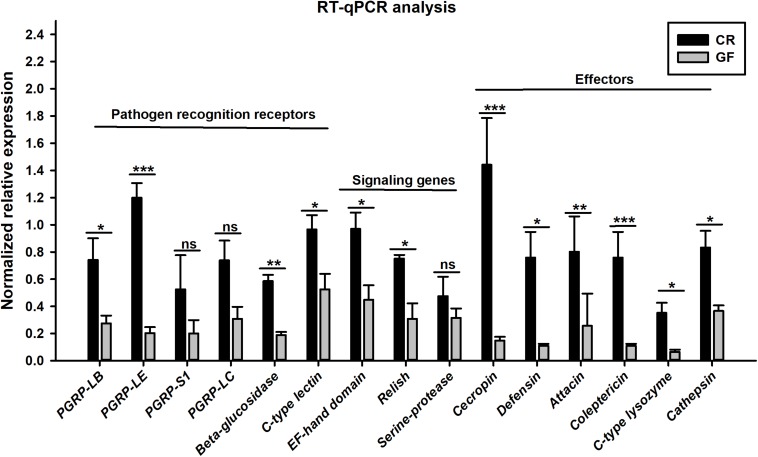
RT-qPCR verification on the transcript abundance of immunity-related genes in the guts of CR and GF RPW larvae. Asterisks indicate statistical difference (*P* < 0.05) between the CR and GF individuals (*t*-test: ns, *P* > 0.05, ^∗^*P* < 0.05, ^∗∗^*P* < 0.01, ^∗∗∗^*P* < 0.001). The fold changes of genes were calculated by normalizing to that of the reference gene, *Rf*β*-actin*, with the 2^–ΔΔCt^ method. Error bars represent the standard deviation of three independent biological replicates.

Two cDNA libraries, corresponding to CR and GF groups, were generated and the Illumina HiSeq^TM^ 4000 system yielded an average of 54,820,970 and 57,311,099 clean reads (total reads 336,396,210), respectively. For in-depth elucidation on the interactions between RPW intestine immunity and its gut microbiota, more than 550 immunity-related genes were determined in the gut transcriptome datasets. These immunity-related genes were classified into the following functional categories such as immune recognition, signal transduction and signal modulation (signaling pathways), immune effectors and others (Supplementary Table S1). To determine the effect of gut commensal microbiota on the expression level of immunity-related genes in this pest, pairwise comparisons were performed between the two libraries to reveal the differentially expressed genes (DEGs). Totally, 1,211 unigenes were found to be differentially expressed between CR and GF insects, of which 1,095 unigenes were downregulated and 116 unigenes were upregulated (Figures 4A,B). Interestingly, 35 immunity-related unigenes were found to be differentially expressed in the guts of CR and GF insects. Significant lower expression level of these unigenes was detected in GF insects, indicating the strong association between gut microbiota and RPW immune system. For instance, a number of immune recognition receptors, including 1 *C-type lectin* and 1 *Galectin* unigene, were found to be significantly decreased (FDR value < 0.05) in GF insects (Figure 5A). The expression level of these unigenes, being involved in signal modulation and signal transduction group, such as Imd, Toll-like receptor, TNF receptor, Tubulin, Rapamycin, Son of Sevenless, MAPK, Ras-related protein, Serine protease, Calmodulin, and EF-hand domain containing protein (total 23 signaling genes), were also drastically impaired in GF individuals (Figure 5B). Some other immune related genes, including *Caspases* and *Dicer*, also presented in GF insects with significantly decreased transcript abundance (Figure 5A). Moreover, the abundance of effector genes, including C-type lysozyme and antimicrobial peptides, were reduced by removing intestinal microbiota from RPW larvae. To confirm these results from RNA sequencing, the transcripts abundance of 15 immune genes was quantified by RT-qPCR. As expected, these genes were expressed in GF insects with significantly lower transcript level as compared to CR ones. For example, the expression level of pathogen recognition receptors, such as *RfPGRP-LB, RfPGRP-LE, RfBeta-glucosidase*, and *RfC-type lectin*, was significantly decreased in GF insects. Both *Relish* and *EF-*hand domain containing protein genes that mediate the immune signal transduction were significantly compromised by the absence of gut microbiota. Additionally, the immune effector genes, such as *RfDefensin, RfAttacin, RfColeoptericin, RfCecropin, RfC-type lysozyme*, and *RfCathepsin*, exhibited in GF insects with strikingly lower transcript abundance (Figure 6). Taken together, these findings demonstrate that gut microbiota can stimulate the expression of immune related genes in the gut of RPW to affect host physiology.

### The Symbiotic Insects Exhibit Stronger Magnitude in the Expression Level of Immunity-Related Genes in Fat Body

**FIGURE 7:**
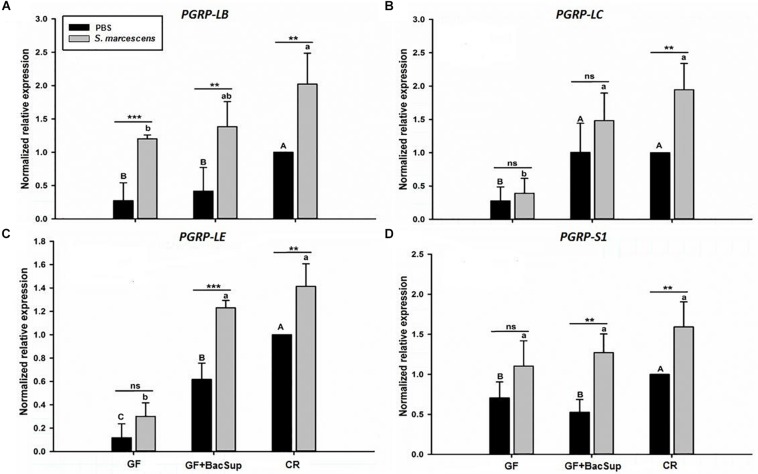
The effect of bacterial challenge on the expression level of four peptidoglycan recognition protein genes in fat body of symbiotic (GF BacSup and CR) and aposymbiotic (GF) RPW larvae. The transcript of PGRP-LB **(A)**, PGRP-LC **(B)**, PGRP-LE **(C)**, and PGRP-S1 **(D)** are evaluated by RT-qPCR in fat body 6 h post-infection with *Serratia marcescens* or PBS as control. One-way ANOVA was conducted to evaluate the statistical differences across the groups using Tukey’s HSD *post hoc* multiple comparison. Uppercase letters indicate the statistical differences of control groups, whereas the lowercase letters represent bacterial infection across the different groups. “^∗^” presents the significance within each cohort (*t*-test: ns, *P* > 0.05, *P* < 0.05, ^∗∗^*P* < 0.01, ^∗∗∗^*P* < 0.001). Error bars represent the standard deviation of four independent replicates.

**FIGURE 8:**
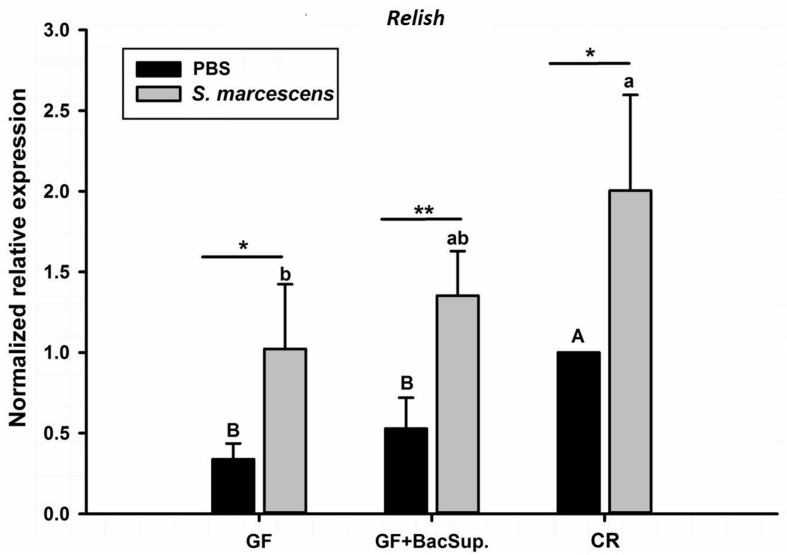
The effect of bacterial challenge on the expression level of *Relish* in the fat body of symbiotic (GF BacSup and CR) and aposymbiotic (GF) RPW larvae. Uppercase and lowercase letters indicate the statistical differences across control and bacteria challenge groups, respectively. “^∗^” presents the significance within each cohort (*t*-test: ^∗^*P* < 0.05, ^∗∗^*P* < 0.01). Error bars represent the standard deviation of four independent replicates.

**FIGURE 9:**
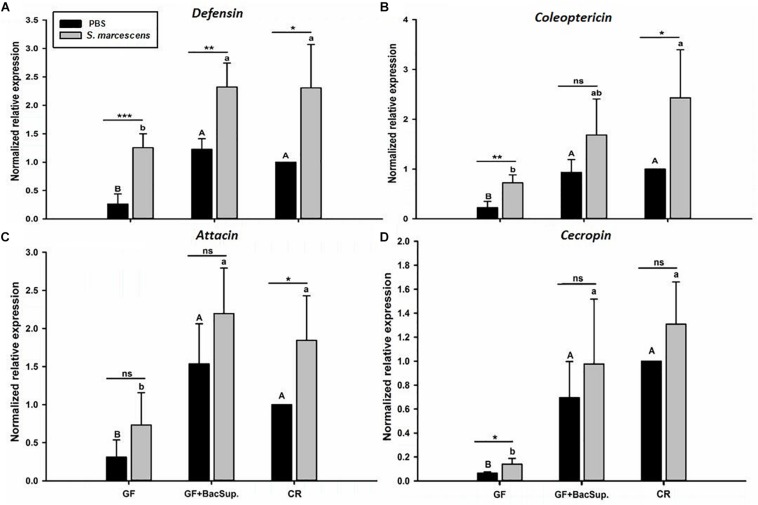
The effect of bacterial challenge on the expression level of antimicrobial peptide genes in fat body of symbiotic (GF BacSup and CKR) and aposymbiotic (GF) RPW larvae. Uppercase and lowercase letters indicate the statistical differences across control and bacteria challenge groups, respectively. “^∗^” presents the significance within each cohort (*t*-test: ns, *P* > 0.05, ^∗^*P* < 0.05, ^∗∗^*P* < 0.01, ^∗∗∗^*P* < 0.001). Error bars represent the standard deviation of four independent replicates. The data was log10 transformed to meet the normality assumptions. The transcript copies of Defensin **(A)**, Coleoptericin **(B)**, Attacin **(C)** and Cecropin **(D)** were determined by RT-qPCR.

Gut microbiota has been shown to enhance the immunocompetence and survival rate of RPW larvae under the challenge of *S*. *marcescens*. However, the mechanisms underlying the enhancement of gut commensal bacteria on RPW systemic immunity were still unknown. Following the infection, the expression profiles of some important immune genes in fat body, including four PGRP genes (*RfPGRP-LB, RfPGRP-LC, RfPGRP-LE*, and *RfPGRP-S1*), *RfRelish*, and four AMP genes (*RfDefensin, RfColeoptericin, RfAttacin*, and *RfCecropin*), were quantified by RT-qPCR. As expected, the GF individuals had decreased expression level of all tested immune genes in both bacterial challenged and non-infected cohorts (Figure 7). Nevertheless, gut bacterial complementation to GF hosts (GF BacSup) exhibited markedly elevated transcript abundance of these tested genes in contrast to GF ones. These results suggested that gut microbiota could enhance the expression level of these PGRP genes in RPW fat body. The challenge of *S. marcescens* triggered the drastically stronger expression level of four PGRP genes in CR and GF BacSup individuals, indicating that the magnitude of induced expression level of these PGRP genes was strongly dependent on the presence of gut microbiota in RPW. Yet this was not the case for that of *RfPGRP-LE, RfPGRP-S1*, and *RfPGRP-LC* in GF individuals. For *RfRelish*, bacterial infection significantly induced its expression level in fat body of RPW larvae in all groups (Figure 8). As expected, *RfDefensin* and *RfColeoptericin* were drastically induced in fat body of aposymbiotic and symbiotic insects by bacterial challenge (Figures 9A,B). But *RfAttacin* was found to be only upregulated upon bacterial infection in CR individuals (Figure 9C) and only GF individuals exhibited significantly elevated expression level of *RfCecropin* (Figure 9D). Taken together, these observations revealed that the presence of gut microbiota could drastically enhance the transcript abundance of immunity-related genes in fat body of RPW larvae and then improve their systemic immunocompetence to provide protection against pathogen.

## Discussion

It has been previously demonstrated that RPW harbors a complex gut microbiota which is involved in the nutrition metabolism (Muhammad et al., 2017; Prosper et al., 2019). We have also verified that the immune system of this pest play the pivotal role in maintaining and modulating the homeostasis of gut commensal microbiota (Dawadi et al., 2018; Xiao et al., 2019). However, the impact of gut microbiota on the immunity of this pest remains elusive. In the present study, it has been determined that the deprival of intestinal microbiota drastically impaired the pathogen clearance and survival ability of RPW larvae after being challenged by *E*. *coli* and *S*. *marcescens*, respectively. Furthermore, RNA sequencing and RT-qPCR analysis verified that the presence of gut microbiota strongly upregulated the immunity-related genes of RPW larvae, containing pathogen recognition receptors, nuclear factor κB *Relish* and antimicrobial peptides, as compared to GF ones. These findings provide the experimental evidence to support that colonization of gut commensal microbiota has the stimulatory effects on the immune system of RPW larvae and enhance the immunocompetence of host. Several studies have revealed that gut microbiome can inhibit the development of *Plasmodium* and other human pathogens in mosquito by upregulating some important immune genes such as *cecropins, defensin*, and *gambicin* (Gonzalez-Ceron et al., 2003; Cirimotich et al., 2011; Ricci et al., 2012). Interestingly, Futo et al. (2016) observed that *T. castaneum* larvae, with significantly lowered microbial load, exhibited decreased survival upon secondary challenge of *Bacillus thuringiensis*, indicating that gut microbiota plays a crucial role in oral immune priming. Recently, it has been shown that the immune system of honey bee *Apis mellifera* can be stimulated by its native gut microbiota (Kwong et al., 2017). Specifically, *Frischella perrara*, a gut bacterium of honey bee, has been verified to cause a strong immune activation and influence gut immunity and homeostasis in the pylorus (Emery et al., 2017). Therefore, our findings, being consistent with these previous reports, highlight that gut commensal bacteria are essential for the development and maintenance of a healthy immune system in insects.

This study uncovered that the presence of gut bacteria could significantly improve the transcript abundance of several pattern recognition receptors (PRRs) and antimicrobial peptides in fat body. Furthermore, RPW larvae without gut bacteria exhibited the lower ability to clear pathogenic bacteria in hemolymph, impaired PO activity and died at faster rate after microbial challenge. It is well known that insect immunity is often mounted through recognizing invaded pathogens by PRRs and then immune signals are transduced via NF-κB pathway to induce the secretion of effectors (Lemaitre and Hoffmann, 2007; Hetru and Hoffmann, 2009). In this context, our data suggest that gut microbiota can enhance the immunity of RPW larvae via the upregulation of immune genes to stimulate antimicrobial peptide secretion. Notably, our previous investigation has determined that enzymatic PGRP-LB acts as a negative regulator on intestinal immunity to maintain the homeostasis of RPW gut microbiota (Dawadi et al., 2018). Our analysis found RPW PGRP-LB gene was significantly upregulated by the colonization of gut microbiota, suggesting that gut commensal bacteria are also involved in the modulation of RPW immunohomeostasis by elevating the expression level of some immunity negative regulators to avoid over-activation of mucosal immunity. RPW gut is colonized by multiple bacterial species which exert different roles on host fitness (Muhammad et al., 2017; Prosper et al., 2019). Recent studies on the immunomodulatory impacts of commensal bacteria on mammals have identified that these effects are specific for individual bacterium, groups of bacteria or specific components of the microbiota (Hooper et al., 2012; Ivanov and Honda, 2012). In this way, RPW gut bacterial composition may influence the type and robustness of host immune responses. Here we just determined the stimulatory effects of intestinal microbiota on RPW immunity by removing the entire commensal microbiota. Therefore, the possible specific roles of individual bacterium or groups of bacteria on the immune system of RPW deserve further investigation through making the gnotobiotic RPW larvae.

In the present investigation, we also provided the evidence to show that gut microbiota do not only affect gut immunity but also mediate the systemic immune response of RPW larvae. To our best knowledge, it is still unknown on the mechanisms underlying gut commensal bacteria modulate the immune system of insect hosts although the immunostimulatory effects of gut microbiome have been determined. Mounting evidence uncovered that gut microbiome can produce numerous bioactive compounds, containing short chain fatty acids (SCFAs), choline metabolites and lipids, that are important for host physiology (Nicholson et al., 2012; Lee and Hase, 2014; Qin and Wade, 2017). In particular, recent reports on mammals have pointed out that SCFAs have important metabolic functions and are crucial for intestinal health (Canfora et al., 2015). For instance, the SCFAs, containing butyrate, propionate, and acetate, promote intestinal epithelial barrier function and regulate the host mucosal immune system via promoting some specific histone post-translational modifications (Vinolo et al., 2011; Krautkramer et al., 2017; Fellows et al., 2018; Schulthess et al., 2019). In *D. melanogaster*, it has been found that axenic flies have altered insulin signaling and lipid metabolism, and this can be reversed by the provision of a gut microbial metabolite, acetate (Shin et al., 2011; Hang et al., 2014). More importantly, SCFAs can affect mammal cellular and modulate immune responses, in part through affecting gene expression and the epigenome by inhibiting histone deacetylases (Koh et al., 2016; Krautkramer et al., 2016). These reports imply that gut microbiota-derived metabolites act as critical messengers in the crosstalk between microbes and hosts. Consequently, the stimulatory effects of commensal bacteria on the systemic immune responses of RPW larvae might be executed by their fermentative metabolites. The diversity and composition of gut microbiota in insect pests have been studied for over a decade, but the causal relationship among microbe-derived gut metabolites, host signaling pathways and host physiology are still poorly understood outside *D*. *melanogaster*. It is well known that intestine commensal bacteria can release peptidoglycan and uracil to induce the expression of AMPs and ROS (Paredes et al., 2011; Lee and Brey, 2013; Dawadi et al., 2018; Xiao et al., 2019). Therefore, another possibility is that gut bacteria-derived peptidoglycan can pass through gut barrier and enter the hemolymph acting as a ligand for NF-κB activation in fat body. Therefore, the exact mechanisms behind commensal bacteria affect the immunity of RPW larvae need extensive dissections by detecting the behavior of microbe-derived metabolites on host physiology.

In conclusion, our results demonstrated that the presence of gut microbiota could drastically improve the survival rate of bacteria challenged RPW larvae via upregulating the important immune genes to enhance host immunocompetence, suggesting that gut bacteria have strong stimulatory effects on the immune system of RPW larvae. Consequently, we found that this pest relies on its intestinal bacteria for its proper immune functions. Because the innate immune responses are the sole way for insect pests to fight off pathogens, harnessing the ability of intestinal microbiota to affect host immunity can be considered as a very promising pest management tactic. On the other hand, our study set a firm foundation to deeply dissect the interplay between RPW and its gut microbiota.

## Data Availability Statement

The datasets (PRJNA546480) for this study can be accessed from NCBI SRA (https://www.ncbi.nlm.nih.gov/Traces/study/?acc=PRJNA546480) under the accession number (SRR9637668–SRR9637673).

## Author Contributions

ZS conceived and designed the research. AM, PH, and TJ completed the experiments. AM and ZS analyzed the data. ZS, AM, and YH wrote the manuscript. All authors have read and approved the final manuscript.

## Conflict of Interest

The authors declare that the research was conducted in the absence of any commercial or financial relationships that could be construed as a potential conflict of interest.

## Footnotes

1 http://www.ncbi.nlm.nih.gov/BLAST/

## Supplementary Material

FIGURE S1 The number of RPW gut bacterial colony-forming uints (CFUs) from four designated groups.
