# Supplementary figures and images for: Intestinal Microbiota Confer Protection by Priming the Immune System of Red Palm Weevil Rhynchophorus ferrugineus Olivier (Coleoptera: Dryophthoridae)
Source: Front Physiol. 2019 Oct 16;10:1303. doi: 10.3389/fphys.2019.01303 (PMC6805723; doi:10.3389/fphys.2019.01303)

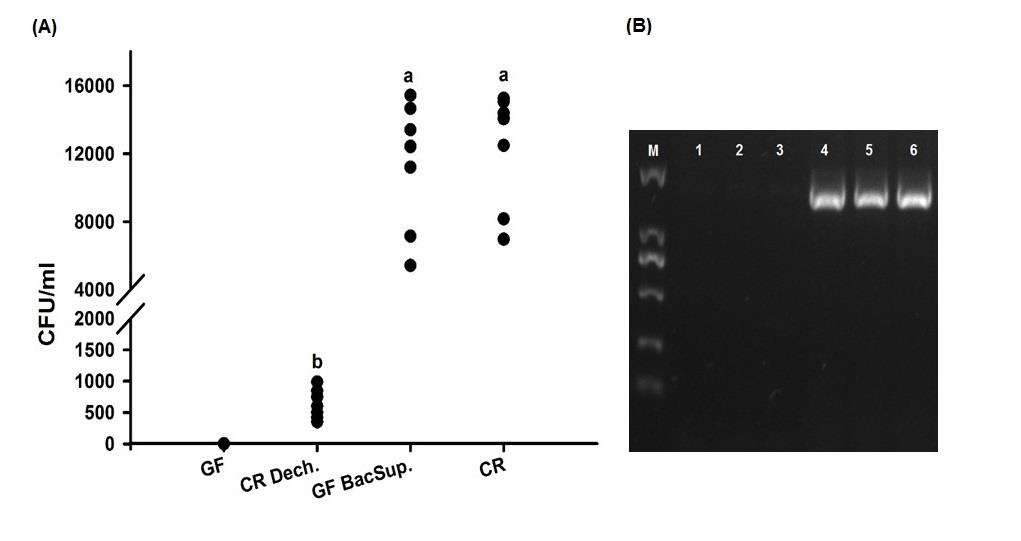

Supplement: FIGURE S1 — The number of RPW gut bacterial colony-forming uints (CFUs) from four designated groups. [file Image_1.JPEG]
